# Ultrasonographic assessment reveals detailed distribution of synovial inflammation in Blau syndrome

**DOI:** 10.1186/ar4533

**Published:** 2014-04-08

**Authors:** Kei Ikeda, Naotomo Kambe, Syuji Takei, Taiji Nakano, Yuzaburo Inoue, Minako Tomiita, Natsuko Oyake, Takashi Satoh, Tsuyoshi Yamatou, Tomohiro Kubota, Ikuo Okafuji, Nobuo Kanazawa, Ryuta Nishikomori, Naoki Shimojo, Hiroyuki Matsue, Hiroshi Nakajima

**Affiliations:** 1 Department of Allergy and Clinical Immunology, Chiba University Hospital, 1-8-1 Inohana, Chuo-ku, Chiba 260-8677, Japan; 2 Department of Dermatology, Chiba University Graduate School of Medicine, 1-8-1 Inohana, Chuo-ku, Chiba 260-8670, Japan; 3 Division of Pediatric Rheumatology, Pediatric Medical Center, Kagoshima University Hospital, 8-35-1 Sakuragaoka, Kagoshima, Kagoshima 890-8520, Japan; 4 School of Health Sciences, Faculty of Medicine, Kagoshima University, 8-35-1 Sakuragaoka, Kagoshima, Kagoshima 890-8520, Japan; 5 Department of Pediatrics, Chiba University Graduate School of Medicine, 1-8-1 Inohana, Chuo-ku, Chiba 260-8670, Japan; 6 Department of Allergy and Rheumatology, Chiba Children’s Hospital, 579-1 Hetacho, Midori-ku, Chiba 266-0007, Japan; 7 Department of Pediatrics, Hitachinaka General Hospital, 20-1 Ishikawacho, Hitachinaka, Ibaraki 312-0057, Japan; 8 Department of Pediatrics, Kobe City Medical Center General Hospital, 2-1-1 Minatojima-minamimachi, Chuo-ku, Kobe, Hyogo 650-0047, Japan; 9 Department of Dermatology, Wakayama Medical University, 811-1 Kimiidera, Wakayama, Wakayama 641-0012, Japan; 10 Department of Pediatrics, Kyoto University Graduate School of Medicine, Yoshida-Konoe-cho, Sakyo-ku, Kyoto 606-8501, Japan

## Abstract

**Introduction:**

Arthritis is the most frequent manifestation of Blau syndrome, an autoinflammatory disorder caused by the genetic mutation of *NOD2*. However, detailed information on arthritis in Blau syndrome on which the therapeutic strategy should be based on is lacking. This multi-center study aimed to accurately characterize the articular manifestation of Blau syndrome and also to demonstrate the utility of musculoskeletal ultrasound in Blau syndrome.

**Methods:**

Patients who had been diagnosed with Blau syndrome by genetic analysis of *NOD2* were recruited. A total of 102 synovial sites in 40 joints were assessed semiquantitatively by ultrasound for gray-scale synovitis and synovial power Doppler (PD) signal.

**Results:**

In total, 10 patients whose age ranged from 10 months to 37 years enrolled in this study. Although only 4 joints (0.8%) were tender on physical examination, 81 joints (16.9%) were clinically swollen. Moreover, 240 (50.0%), and 124 (25.8%) joints showed gray-scale (GS) synovitis and synovial PD signal on ultrasound, respectively. Importantly, GS synovitis was present in 168 out of 399 non-swollen joints, in which 61 also exhibited synovial PD signal. Among 40 joint regions, the ankle, the wrist, and the proximal interphalangeal joints were the most frequently and severely affected joints. Comparisons between different synovial tissues demonstrated a significantly higher proportion of the joints with tenosynovitis as compared with that with intra-articular synovitis (41.5% versus 27.9%, *P* < 0.0001). In respect of age and treatment, synovial PD signals were minimal in the youngest patient and in the oldest two patients, and were relatively mild in patients receiving treatment with methotrexate plus TNF antagonists. In two patients who underwent the second ultrasound examination, total PD scores markedly decreased after initiating the treatment with a tumor necrosis factor (TNF) antagonist.

**Conclusions:**

The detailed information on synovial inflammation obtained by ultrasound confirms the dissociation between pain and inflammation and the frequently involved joint regions and synovial tissue in the arthritis of Blau syndrome. Our data also demonstrate that ultrasonography can be a potent tool in monitoring the activity of synovial inflammation and in investigating the pathophysiology of arthritis in this rare but archetypical autoinflammatory condition.

## Introduction

Blau syndrome (MIM #186580) is a rare autoinflammatory disorder, which was first described in 1985 by the pediatrician Edward Blau as a dominantly inherited, chronic inflammatory syndrome characterized by the clinical triad of granulomatous dermatitis, symmetric arthritis and recurrent uveitis [1]. In 2001, Miceli-Richard *et al*. identified the gene that confers susceptibility for Blau syndrome, discovering three mis-sense mutations (R334Q, R334W and L469F) in the region encoding the nucleotide-binding oligomerization domain (NOD) of the caspase recruitment domain gene (*CARD15/NOD2*) in four French and German affected families [2]. In spite of the striking clinical similarities with Blau syndrome, early onset sarcoidosis (EOS, MIM #609464) was originally considered a distinct disease entity. However, subsequent genetic analyses have shown that many patients with EOS have mutations in *NOD2*[3-6] and these two diseases and other variant forms are now considered to represent a pathophysiologically identical condition in which increased NFκB activity driven by mutated *NOD2* gene plays a significant role [3,6-11].

Arthritis is the most frequent manifestation of Blau syndrome and usually becomes clinically apparent within the first decade of life [1,5,7,11-15]. Joint manifestation in Blau syndrome has been reported to be chronic, symmetrical, and mostly painless polyarthritis. Marked soft-tissue swelling can occur due to granulomatous inflammation in both intra-articular synovium and tenosynovium, which can cause characteristic camptodactyly (that is, flexion contracture of fingers and toes) and, occasionally, subsequent impairment of physical function. However, the precise location and severity of joint inflammation in patients with Blau syndrome have not been systematically evaluated because of the difficulty to accurately determine with physical examination the activity of current inflammation in mostly non-tender joints in children.

Musculoskeletal ultrasound is a relatively inexpensive imaging modality, which enables more accurate assessment of synovial inflammation than physical examination does [16-19] and, therefore, provides improved accuracy in the diagnosis [20-24] and the disease activity monitoring [19,23-29] of rheumatoid arthritis (RA). As ultrasound is a non-invasive technique, which patients can undergo with minimal restriction, its use in pediatric inflammatory conditions such as juvenile idiopathic arthritis (JIA) has been increasingly studied [30-33]. We recently reported representative ultrasound images that clearly visualized the tenosynovitis in a patient with Blau syndrome [34]; however, no other papers have reported the use of ultrasound in the assessment of synovial inflammation in Blau syndrome.

In this pilot, multicenter study, 10 patients with Blau syndrome whose *NOD2* mutation had been confirmed underwent comprehensive ultrasound examination of 102 synovial sites, aiming to accurately characterize the articular manifestation of Blau syndrome and also to demonstrate the utility of musculoskeletal ultrasound in Blau syndrome.

## Patients and methods

### Patients and *NOD2* mutation analysis

In this cross-sectional study, patients who had been diagnosed with Blau syndrome by genetic analysis of *NOD2* were recruited. Age- and sex-matched control subjects who did not have arthritic symptoms were also recruited. Written informed consent was obtained from the patient/subject, or his/her family member if necessary, according to the study protocol approved by the Ethics Committee of Chiba University, Kyoto University, and Kagoshima University in accordance with the Declaration of Helsinki. Genomic DNA was extracted from the peripheral blood of the patients, and sequencing of all exons and exon-intron junctions of *NOD2* was performed as previously described [6].

### Clinical and laboratory assessment

Clinical information collected was complete medical history including current and previous medication for Blau syndrome, tender and swollen joint counts in 40 joints (disease activity score in 28 joints (DAS28) + bilateral ankle and metatarsophalangeal (MTP) joints), patient’s/parent’s and physician’s global visual analogue scale (VAS), erythrocyte sedimentation rate (ESR), serum levels of C-reactive protein (CRP) and matrix metalloproteinase-3 (MMP-3), childhood health assessment questionnaire-disability index (CHAQ-DI), and the presence of camptodactyly in fingers and toes.

### Ultrasound examination

A systematic multiplanar gray-scale (GS) and power Doppler (PD) ultrasound was performed in a temperature-controlled room on the same day of the clinical evaluation by a rheumatologist who was experienced in musculoskeletal ultrasound (KI) using an HI VISION Ascendus with a linear array multi-frequency transducer (5 to 18 MHz for GS, 7.5 MHz for PD) (Hitachi Medical Corporation, Tokyo, Japan). PD ultrasound was performed with a pulse repetition frequency set at 800 Hz and a low wall filter. Color gain was set just below the level at which color noise appeared.

**Figure 1:**
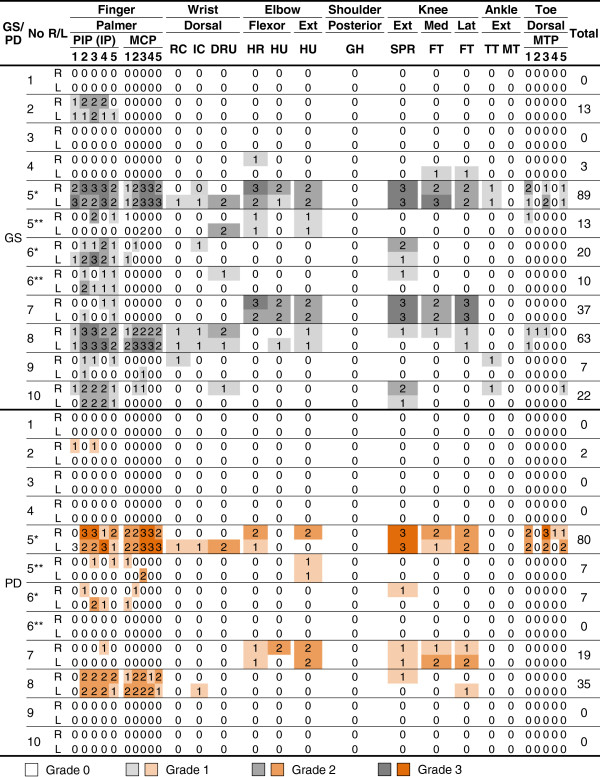
**Ultrasound scores for intra-articular synovitis in patients with Blau syndrome.** Semiquantitative grades (0 to 3) for gray-scale synovitis and synovial power Doppler signal in individual joint regions are shown in heat maps. Total sum of the scores in each case is shown in the right column. *First ultrasound examination (before treatment); **second ultrasound examination (after treatment). GS, gray-scale; PD, power Doppler; No, case number; R, right; L, left; Ext, extensor; Lat, lateral; Post, posterior; Med, medial; PIP (IP), proximal interphalangeal (interphalangeal); MCP, metacarpophalangeal; RC, radiocarpal; IC, intercarpal; DRU, distal radioulnar; HR, humeroradial; HU, humeroulnar; GH, glenohumeral; SPR, suprapatellar recess; FT, femorotibial; TT, tibiotalar; IT, intertarsal; MTP, metatarsophalangeal.

**Figure 2:**
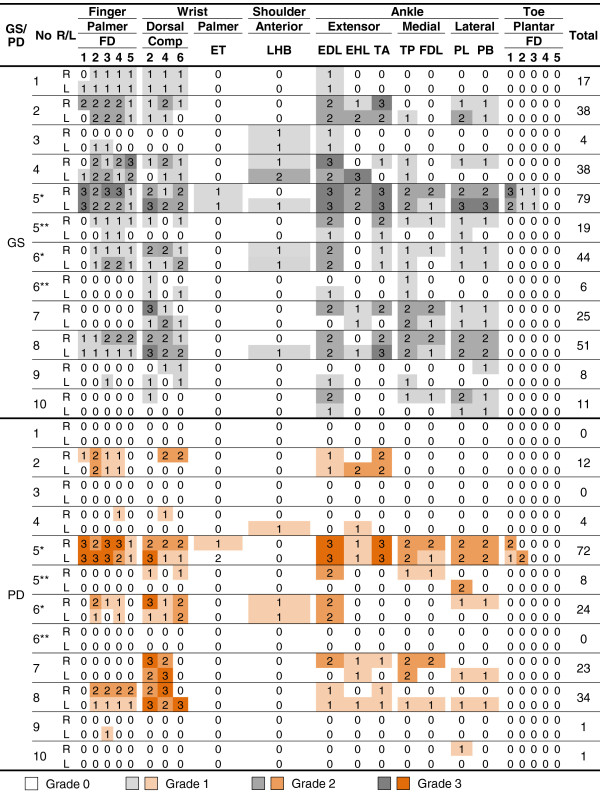
**Ultrasound scores for tenosynovitis in patients with Blau syndrome.** Semiquantitative grades (0 to 3) for gray-scale synovitis and synovial power Doppler signal in individual joint regions are shown with heat maps. Total sum of the scores in each case is shown in the rightmost column. *First ultrasound examination (before treatment); **second ultrasound examination (after treatment). GS, gray-scale; PD, power Doppler; No, case number; R, right; L, left; FT, flexor tendon; Comp, compartment; ETs, extensor tendons (flexor digitorum superficialis/profundus and flexor carpi radialis); LHB, long head of biceps tendon; EDL, extensor digitorum longus; EHL, extensor hallucis longus; TA, tibialis anterior; TP, tibialis posterior; FDL, flexor digitorum longus; PL, peroneus longus; PB, peroneus brevis.

Ultrasound was performed on the 40 joints which were clinically assessed for joint counts. Middle and large joints were divided into individual joints which compose the larger joint unit (for example, humeroradial and humeroulnar joints in the elbow) or into multiple parts of the joint (for example, suprapatellar recess, medial and lateral aspects of femorotibial joints in the knee) and a total of 54 intra-articular synovial sites were separately evaluated (Figure 1). In addition to the intra-articular synovium, we also evaluated the major peri-articular synovial tissues; a total of 44 tenosynovial sites (Figure 2) and 4 bursal sites (bilateral sub-acromial/deltoid bursae) were examined. All sites were scanned thoroughly in multiple imaging planes so that the whole lesion was evaluated. As no ultrasound definitions of synovial pathology have been established for children, we based our assessment on the ultrasound definitions for adults [35]. However, particularly careful attention was paid in children to distinguishing unossified cartilage and its feeding blood vessels from true synovial pathology. As no ultrasound scoring system for synovial pathology in children has been established either, severity of synovial pathology in the 102 sites was graded subjectively utilizing the rater’s experience in the clinical studies in adults [19,22,29,36-38]. Synovial hypertrophy and synovial fluid were graded as a GS score altogether on a scale of 0 to 3 (grade 0, normal; grade 1, mild; grade 2, moderate; grade 3, severe) based on the rater’s impression of how large the volume of synovial hypertrophy and synovial fluid was for the synovial site assessed. On the other hand, synovial PD signal was graded as a PD score on a scale of 0 to 3 (grade 0, normal; grade 1, mild; grade 2, moderate; grade 3, severe) based on the rater’s impression of how widespread the abnormal synovial PD signals within the synovial hypertrophy were. Total scores for an individual patient were calculated for each synovial tissue and for all synovial tissues by summation for each synovial tissue (that is, total intra-articular GS/PD score, total tenosynovial GS/PD score, total bursal GS/PD score) and for all synovial tissues (that is, total synovial GS/PD score). When ultrasound findings were assessed at a 40-joint level, the maximum grade obtained from multiple synovial sites (for example, radiocarpal joint, extensor carpi ulnaris) within a joint unit was assigned to the joint (for example, wrist).

### Statistical analysis

Statistical analysis was performed using R version 2.13.0 (The R Foundation for Statistical Computing, Vienna, Austria). Non-normally distributed continuous data were analyzed using nonparametric tests (Spearman’s rank correlation coefficient and Wilcoxon signed-rank test). Categorical data were analyzed using McNemar’s test. Bonferroni’s correction was applied for multiple testing. *P*-values less than 0.05 were considered statistically significant.

## Results

### Demographics and disease characteristics

Ten patients (two female) from eight unrelated Japanese families enrolled in this study. As shown in Table 1, the age at ultrasound examination ranged from 10 months to 37 years and the patients were numbered from 1 to 10 in order of this age. Patients 1, 2, and 10 were offspring and a parent, whereas the other patients had no family history suggestive of Blau syndrome. All patients had been shown to have a known *NOD2* gene mutation associated with Blau syndrome (Table 1). Patient 3 was an atypical case who developed aortitis at the age of 7 months, which was treated successfully with corticosteroid (pulse methylprednisolone followed by oral prednisolone for 5 years), methotrexate, and infliximab as infantile Takayasu arteritis. He was genotyped for *NOD2* when he developed iritis at the age of 7 years [39].

**Table 1:** Demographics, disease characteristics, and treatment at ultrasound examination

**Patient number**	***NOD2 *** **mutation**	**Age (months)**				**Duration of treatment at ultrasound examination (months)**			
		**At ultrasound examination**	**At onset of joint symptom**	**At onset of skin lesion**	**At diagnosis of uveitis**	**NSAID**	**Corticosteroid**	**Methotrexate**	**TNF antagonist**
1^†^	R334W	10	NA	6	NA	NA	NA	NA	NA
2^†^ ref [34]	R334W	66	19	6	NA	NA	NA	13	NA
3 ref [36]	D382E	111	NA	NA	88	NA	NA	60	11 (infliximab)
4	R587C	115	53	NA	NA	NA	NA	32	32 (etanercept)
5	R334W	146*	24	7	39	NA	NA	NA	NA
		154**				NA	NA	3	3 (infliximab)
6	R334W	153*	29	21	35	12	112	124	NA
		166**				NA	NA	137	12 (infliximab)
7 ref [6]	R334Q	160	52	NA	NA	105	NA	60	44 (infliximab)
8 ref [6]	R334W	212	24	8	48	188	142	184	NA
9 ref [6]	R334Q	294	34	24	60	270	259	135	45 (infliximab)
10^†^ ref [3]	R334W	452	NR^‡^	24	156	NA	NA	NA	NA

ref: Reference number in which the patient was previously reported; ^†^offspring and parent; *1^st^ ultrasound examination; **2^nd^ ultrasound examination; ^‡^reported no arthritic symptoms despite the presence of overt camptodactyly. NSAID, non-steroidal anti-inflammatory drug; TNF, tumor necrosis factor; NR, not reported; NA, not applicable.

### Treatment at ultrasound examination

Three patients (patients 1, 5, and 10) were not receiving any medication for joint involvement when ultrasound was performed, and seven patients were receiving anti-inflammatory/rheumatic medications, including nonsteroidal anti-inflammatory drugs (NSAIDs), corticosteroids (prednisolone ≤11.25 mg/day), methotrexate, and TNF antagonists (Table 1). In patient 5, treatment with methotrexate plus infliximab was initiated after the first ultrasound examination and the second ultrasound examination was performed after 14 weeks of treatment. In patient 6, infliximab was added to his treatment regimen after the first ultrasound examination and the second ultrasound examination was performed after 12 months.

### Severity of arthritis assessed by conventional measures

Table 2 summarizes the severity of arthritis at ultrasound examination. Although swollen joint counts varied among patients, most of the patients did not have any tender joints and therefore, the patient’s or parent’s global VAS score was ≤10 mm in most of the patients. The levels of acute inflammatory responses and physician’s global VAS score were not markedly elevated either. As shown in Table 2, disease activity parameters in patient 5 substantially improved after treatment with methotrexate plus infliximab, whereas the decrease in those parameters in patient 6 was relatively mild.

**Table 2:** Conventional activity measures for joint inflammation, physical function, and the presence of camptodactyly

**Patient number**	**Tender joint count**		**Swollen joint count**		**Global assessment VAS (/100 mm)**		**Acute inflammatory response (normal range**^§^**)**		**DAS28 -ESR**	**DAS28 -CRP**	**MMP-3**^§§^**(ng/mL)**	**CHAQ**	**Campto- dactyly**
	**/28**	**/40**	**/28**	**/40**	**Patient/parent**	**Physician**	**ESR, mm/h**	**CRP, mg/L**					
1	NR^†^	NR^†^	0	0	0	2	3 (2 to 10)	3.0 (0 to 3)	NA	NA	27.2	0	No
2	0	0	22	24	10	5	6 (2 to 10)	1.0 (0 to 3)	2.7	2.7	85.3	0	No
3	0	0	0	0	0	3	29 (2 to 10)	32.0 (0 to 3)	2.4	2.2	24.9	0	No
4	0	0	1	1	8	6	19 (3 to 15)	0.7 (0 to 2.6)	2.5	1.5	64.0	0.13	Yes
5*	0	0	23	25	10	50	22 (1 to 10)	6.0 (0 to 3)	3.7	3.1	800.0	0	Yes
5**	0	0	7	9	0	23	3 (1 to 10)	0.0 (0 to 3)	1.5	1.7	21.8	0	Yes
6*	0	0	2	3	1	5	3 (5 to 13)	3.0 (0 to 2)	1.3	1.9	18.7	0	Yes
6**	0	0	2	2	2	1	2 (5 to 13)	0.4 (0 to 2)	0.9	1.5	18.8	0	Yes
7	0	0	1	1	0	0	27 (3 to 15)	1.4 (0 to 2.6)	2.6	1.6	426.4	0	Yes
8	4	4	13	13	65	45	8 (2 to 10)	1.0 (0 to 2.6)	4.5	4.3	36.9	0.63	Yes
9	0	0	0	0	15	8	4 (2 to 10)	0.0 (0 to 2.6)	1.2	1.2	216.8	1.63^‡^	Yes
10	0	0	3	5	4	8	12 (2 to 10)	1.0 (0 to 3)	2.3	1.8	129.0	0.13^‡^	Yes

*1^st^ ultrasound examination ; **2^nd^ ultrasound examination; ^†^not reported because the patient was not able to understand and answer the question; ^‡^health assessment questionnaire-damage index (HAQ-DI) for adult was obtained; ^§^normal range in the institute for patient’s sex is provided; ^§§^normal ranges are 36.9 to 121.0 ng/mL for males and 17.3 to 59.7 ng/mL for females. VAS, visual analogue scale; ESR, erythrocyte sedimentation rate; CRP, C-reactive protein; DAS28, disease activity score 28; MMP-3, matrix metalloproteinase-3; CHAQ, childhood-health assessment questionnaire; NR, not reported; NA, not applicable.

**Figure 3:**
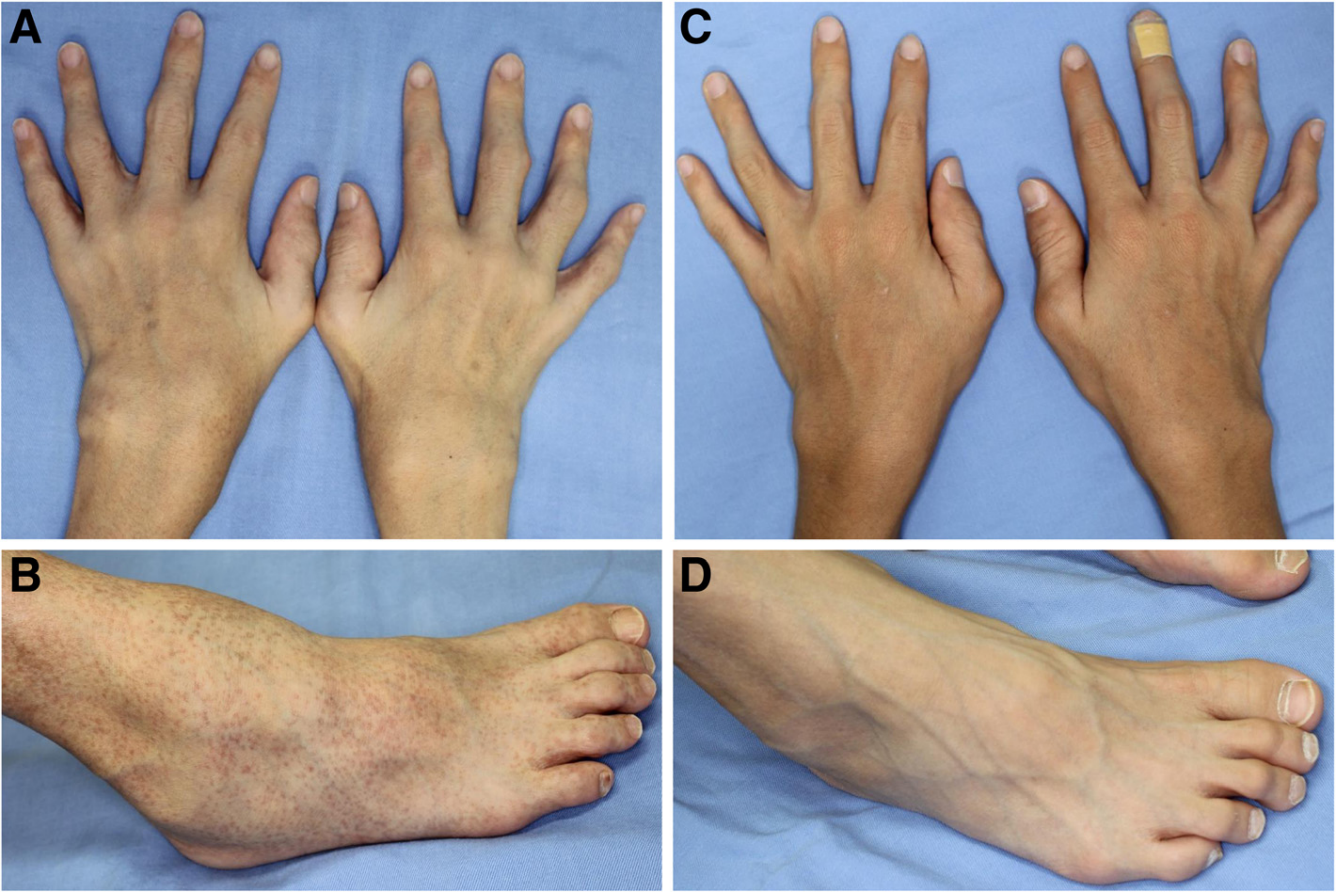
**Photographs of the hands and the right foot in patient 5.** Shown are the photographs of the hands **(A, C)** and the right foot **(B, D)** in Patient 5 before treatment **(A, B)** and after treatment **(C, D)**. Soft tissue swelling in the wrists, the fingers, the ankle, and the mid foot improved after treatment with methotrexate and infliximab. Papular rash also markedly improved.

**Figure 4:**
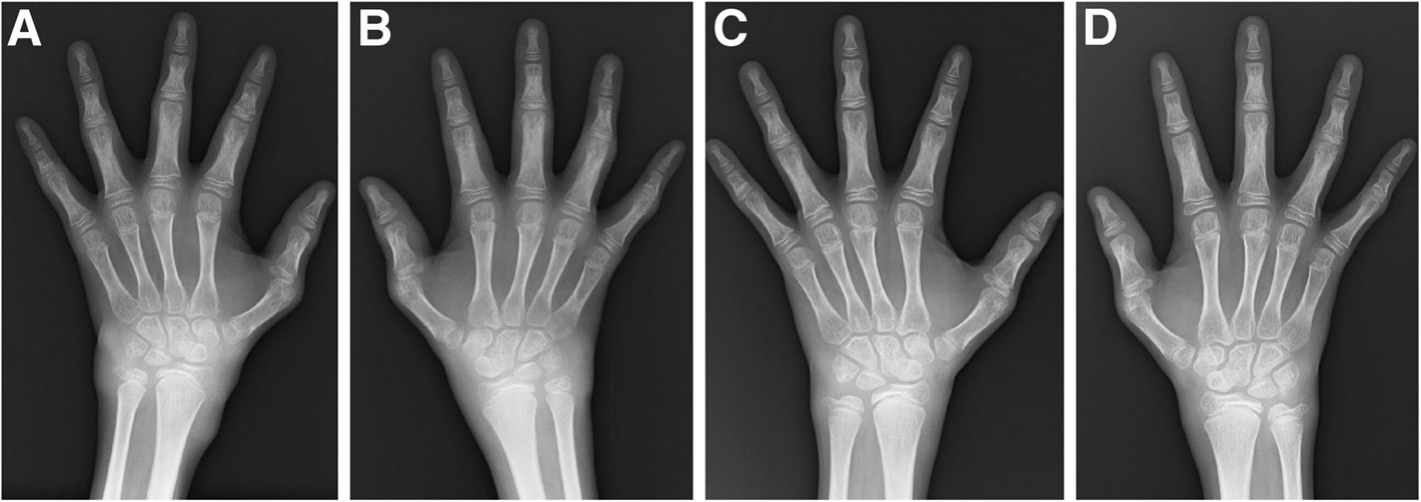
**Radiographs of the hands in patient 5.** Shown are the radiographs of the left hand **(A, C)** and the right hand **(B, D)** in patient 5 before treatment **(A, B)** and after treatment **(C, D)**. Soft tissue swelling in the wrist and fingers improved after treatment with methotrexate and infliximab.

Camptodactyly was present at least in one joint in patients 4 to 10, whose ages were 9 years and 7 months or older (Table 2). Proximal interphalangeal (PIP) joints were the joints in which camptodactyly was identified most frequently. Despite the presence of multiple swollen joints and camptodactyly, physical function assessed by CHAQ/HAQ was rarely impaired in patients younger than 13 years. Photographs of the hands and the right foot and radiographs of the hands in patient 5 are shown in Figures 3 and 4, respectively.

### Distribution of inflammation in synovial tissues determined by ultrasound

All patients completed the comprehensive ultrasound assessment of 102 synovial sites. The distribution and the grade of GS and PD scores in each patient were illustrated in heat maps for intra-articular synovitis (Figure 1) and for tenosynovitis (Figure 2). Bursitis was identified only in the sub-acromial/deltoid bursa in bilateral shoulders in one patient (patient 6) with a GS score of 2 bilaterally and a PD score of 2 in the right shoulder (data not shown).

**Figure 5:**
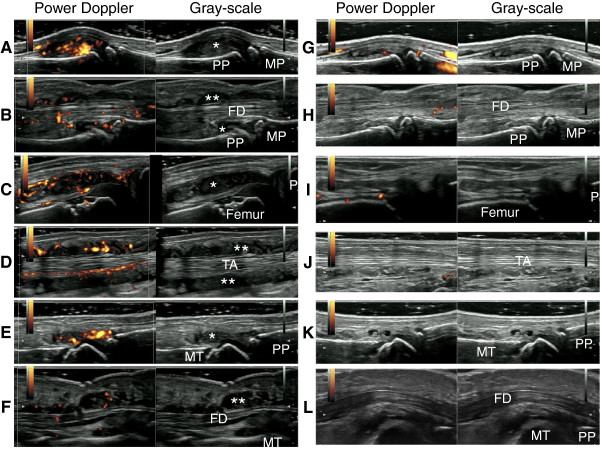
**Representative ultrasound images of intra-articular synovitis and tenosynovitis in patient 5.** Shown are the representative ultrasound images of intra-articular synovitis **(A, C, E, G, I, K)** and tenosynovitis **(B, D, F, H, J, L)** in patient 5 before **(A-F)** and after treatment **(G-L)**. All images before treatment demonstrate synovial/tenosynovial hypertrophy, which accompanies increased power Doppler signals **(A-F)**. Additional movie files show this in more detail (see Additional files 1, 2, 3, 4, 5 and 6). **(A and G)** Longitudinal view of the dorsal aspect of the 3^rd^ proximal interphalangeal joint in the right hand; **(B and H)** longitudinal view of the flexor tendon of the third finger on the right hand; **(C and I)** oblique view of the anterior-lateral aspect of the suprapatellar recess in the right knee; **(D and J)** a longitudinal view of the tibialis anterior tendon in the right ankle; **(E and K)** longitudinal view of the dorsal aspect of the third metatarsophalangeal joint of the right foot; **(F and L)** longitudinal view of the flexor tendon of the first toe of the right foot. *Intra-articular synovial hypertrophy; **tenosynovial hypertrophy. PP, proximal phalanx; MP, metacarpal; FD, flexor digitorum; TA, tibialis anterior; MT, metatarsal.

As demonstrated in Figures 1 and 2, a wide range of synovial sites were affected, mostly in a symmetrical manner. Representative ultrasound images of patient 5 are shown in Figure 5. Additional movie files show these in more detail (see Additional files 1, 2, 3, 4, 5 and 6). The most frequently and severely involved synovial sites were tendon sheaths in the ankle, extensor tendon sheaths in the wrist, and the PIP joints. In contrast, some synovial sites, such as the glenohumeral joint, the midtarsal joint, and the olecranon bursa, did not exhibit synovial inflammation on ultrasound.

Ten age- and sex-matched control subjects, two female, mean age 169.8 months old (range 3 to 499) also underwent comprehensive ultrasound assessment. Only mild GS intra-articular synovitis and tenosynovitis without a PD signal was identified in subjects aged 3 years or older. GS intra-articular synovitis was identified in nine sites in eight joints (three wrists, three toes) in four patients and GS tenosynovitis was identified in five sites in four joints (three ankles) in four patients. These data indicate that GS scores of 2 or greater and positive PD scores have pathological meanings, whereas GS scores of 1 are less specific in our study.

### Predominance of tenosynovitis over intra-articular synovitis

**Figure 6:**
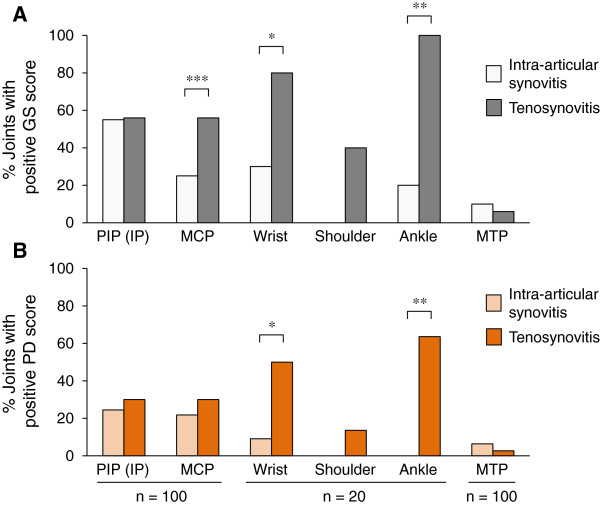
**Prevalence of intra-articular synovitis and tenosynovitis in each joint.** Shown are the comparisons of prevalence of joints with a positive gray-scale **(A)** or power Doppler **(B)** score between intra-articular synovitis (light gray/orange bar) and tenosynovitis (dark gray/orange bar) in each joint. Numbers of total joints assessed for the region are shown in the bottom (n). *P <0.05, **P <0.01, ***P <0.001, McNemar test with Bonferroni correction. GS, gray-scale; PD, power Doppler; PIP (IP), proximal interphalangeal (interphalangeal) joint; MCP, metacarpophalangeal joint; MTP, metatarsophalangeal joint.

Tenosynovitis was more prevalent and more severe than intra-articular synovitis in our patients with Blau syndrome (Figures 1 and 2). When compared in the total joints where both intra-articular- and teno-synovitis were evaluated (that is, elbow and knee joints were excluded), the proportion of the joints with a positive GS score for tenosynovitis was significantly higher than that for intra-articular synovitis (41.5% versus 27.9%, *P* <0.0001, McNemar test with Bonferroni correction). When compared in each joint, the prevalence of GS tenosynovitis was significantly higher than that of GS intra-articular synovitis in the metacarpophalangeal (MCP) joints (56.0% versus 25.0%, *P* <0.0001), in the wrist (80.0% versus 30.0%, *P* = 0.0266), and in the ankle (100% versus 20.0%, *P* = 0.0011) (McNemar test with Bonferroni correction) (Figure 6A). Similarly, the proportion of the joints with a positive PD score for tenosynovitis was significantly higher than that for intra-articular synovitis in the total joints (22.2% versus 15.2%, *P* <0.0001), and individually, in the wrist (55.0% versus 15.0%, *P* = 0.0460) and in the ankle (60.0% versus 0.0%, *P* = 0.0090) (McNemar test with Bonferroni correction) (Figure 6B). In addition, the GS and PD scores for tenosynovitis were significantly higher than those for intra-articular synovitis in the total joints (*P* <0.0001 for both scores, Wilcoxon signed-rank test with Bonferroni correction).

### Improvement of ultrasound findings after initiating the treatment with infliximab

In patient 5, clinical, radiographic, and ultrasonographic findings markedly improved after 14 weeks of treatment with methotrexate plus infliximab (total intra-articular GS score, 89 versus 13; total intra-articular PD score, 80 versus 7; total tenosynovial GS score, 79 versus 19; total tenosynovial PD score, 72 versus 8) (Figures 1, 2, 3, 4 and 5). This result suggests that treatment with methotrexate plus infliximab can improve the inflammation in synovial tissues in a patient with Blau syndrome in a relatively short period of time.

Adding infliximab to methotrexate also substantially improved the ultrasound findings in patient 6, leaving no residual PD signals in the synovial tissues even after the corticosteroid and the NSAID were discontinued (total intra-articular GS score, 20 versus 10; total intra-articular PD score, 7 versus 0; total tenosynovial GS score, 44 versus 6; total tenosynovial PD score, 24 versus 0) (Figures 1 and 2). These data indicate that ultrasound scores are more responsive to change as compared with conventional measures.

### Influence of age and treatment on inflammation in synovial tissues

Also demonstrated in Figures 1 and 2, together with Table 1, is the influence of age and treatment on synovial inflammation. Focusing on the PD score, severe cases of intra-articular synovitis clustered between patient 5 (12 years and 2 months old) and patient 8 (18 years and 8 months old) (Figure 1). On the other hand, severe cases of tenosynovitis clustered between patient 2 (5 years and 6 months old) and patient 8 (Figure 2). Of note, ultrasound revealed little or no inflammation accompanying the PD signal in patient 1 (10 months old) and patient 10 (37 years and 8 months old) even though they had never received anti-inflammatory or rheumatic treatment, whereas patient 2, who is a member of the same family carrying the same *NOD2* mutation, exhibited substantial PD tenosynovitis despite receiving an NSAID and methotrexate. Patients within the cluster whose ultrasound images showed relatively mild PD signals (patients 3, 4, 5 (after treatment) and 6 (after treatment)) were all receiving treatment with methotrexate plus a TNF antagonist.

### Discrepancy between ultrasound scores and conventional measures for the activity of arthritis

As shown in Table 3, the swollen joint count tended to correlate with total ultrasound scores (ρ 0.68 to 0.76, *P* 0.0364 to 0.1343). However, no other conventional measures for arthritis activity correlated with total ultrasound scores with statistical significance.

**Table 3:** Correlation between total ultrasound scores and conventional activity measures

		**Tender joint count /40**	**Swollen joint count /40**	**Patient’s VAS**	**Physician’s VAS**	**ESR**	**CRP**	**DAS28 -ESR**	**DAS28 -CRP**	**MMP-3**
**Intra-articular synovium**	**Total GS score**	0.30	0.76*	0.36	0.51	0.21	0.11	0.53	0.55	0.19
	**Total PD score**	0.35	0.69	0.18	0.40	0.20	0.25	0.63	0.61	0.45
**Tenosynovium**	**Total GS score**	0.00	0.68	0.42	0.47	0.13	0.21	0.67	0.57	0.45
	**Total PD score**	0.55	0.72	0.40	0.51	0.23	0.17	0.66	0.59	0.30
**Total**	**Total GS score**	0.20	0.72	0.34	0.39	0.21	0.28	0.67	0.59	0.33
	**Total PD score**	0.37	0.68	0.35	0.51	0.27	0.12	0.66	0.53	0.25

Values are Spearman’s correlation coefficients (ρ). *P < 0.05; no other correlations were statistically significant when Bonferroni correction was applied. VAS, visual analogue scale; ESR, erythrocyte sedimentation rate; CRP, C-reactive protein; DAS28, disease activity score 28; MMP-3, matrix metalloproteinase-3; GS, gray-scale; PD, power Doppler.

At a 40-joint level, numbers of joints with tenderness, swelling, GS ≥1 synovitis (GS score ≥1), GS ≥2 synovitis (GS score ≥2), and synovial PD signal were 4 (0.8%), 81 (16.9%), 240 (50.0%), 117 (24.4%), and 124 (25.8%), respectively. The differences in prevalence between swelling and ultrasound findings were statistically significant (swelling versus GS ≥1 synovitis, *P* <0.0001; swelling versus GS ≥2 synovitis, *P* = 0.0002; swelling versus synovial PD signal, *P* <0.0001) (McNemar test with Bonferroni correction). Importantly, GS ≥1/GS ≥2 synovitis was present in 168/57 out of 399 non-swollen joints, in which 61 also exhibited synovial PD signal (Table 4).

**Table 4:** Discrepancy between joint swelling and ultrasound findings

		**Swelling**		**Total**
		**(−)**	**(+)**	
**Gray-scale synovitis**	**(−)**	231 (48)	9 (2)	240 (50)
**(gray-scale score ≥1)**	**(+)**	168 (35)	72 (15)	240 (50)
**Total**		399 (83)	81 (17)	480 (100)
**Gray-scale synovitis**	**(−)**	342 (71)	21 (4)	363 (75)
**(gray-scale score ≥2)**	**(+)**	57 (12)	60 (13)	240 (25)
**Total**		399 (83)	81 (17)	480 (100)
**Synovial**	**(−)**	338 (70)	18 (4)	356 (74)
**Power Doppler signal**	**(+)**	61 (13)	63 (13)	124 (26)
**Total**		399 (83)	81 (17)	480 (100)

Values are the number (%) of joint regions.

**Figure 7:**
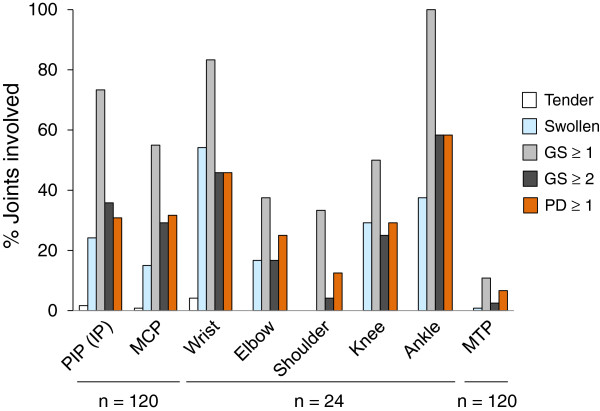
**Prevalence of clinical manifestations and ultrasound findings in each joint.** Shown are the proportions of joints with tenderness (white bar), swelling (light blue bar), gray-scale synovitis ≥ grade 1 (light gray bar), gray-scale synovitis ≥ grade 2 (dark gray bar), and synovial power Doppler signal (orange bar). Numbers of total joints assessed for the region are shown in the bottom (n). **P <0.01, ***P <0.001, McNemar test with Bonferroni correction. GS, gray-scale score; PD, power Doppler score; PIP (IP), proximal interphalangeal (interphalangeal) joint; MCP, metacarpophalangeal joint; MTP, metatarsophalangeal joint.

In each joint region, prevalence of GS ≥1 synovitis was significantly higher than that of joint swelling in the PIP joints (73.3% versus 24.2%, *P* <0.0001), the MCP joints (55.0% versus 15.0%, *P* <0.0001), and the ankle (100% versus 37.6%, *P* = 0.0072) (McNemar test with Bonferroni correction) (Figure 7). Prevalence of GS ≥1 synovitis and synovial PD signal were also significantly higher than that of joint swelling in the MCP joints (29.2% and 31.7% versus 15.0%, *P* = 0.0058 and *P* = 0.0012, respectively, McNemar test with Bonferroni correction) (Figure 7).

## Discussion

In this multi-center study, ultrasonographic assessment of 102 synovial sites provided detailed information on the anatomical distribution of synovial inflammation in patients with Blau syndrome. Our data demonstrate that a wide range of synovial sites can be affected mostly in a symmetrical manner in patients with Blau syndrome. Most frequently involved joints identified in this study are consistent with the previous reports [1,7,13-15]. However, our data provide novel and more specific information on the anatomical site (intra-articular synovium versus tenosynovium versus bursa), the chronicity (currently active inflammation versus chronic swelling versus subsequent contracture), and the severity (mild versus severe). As a result, the data in the form of a heat map (Figures 1 and 2) readily provide clinicians and researchers with valuable information. Furthermore, our data for the first time demonstrate the predominance of tenosynovitis over intra-articular synovitis in Blau syndrome. Although frequent involvement of the tenosynovium has been reported descriptively [1,7,13-15], no studies had provided objective data on the predominance of tenosynovitis.

Although this is a cross-sectional study except for patient 5 and 6, comprehensive and detailed data from patients at a wide range of ages who share the same underlying molecular pathophysiology let us speculate on the natural history of synovial inflammation in Blau syndrome. Comparing the siblings (patients 1 and 2), we assume that mild GS tenosynovitis precedes clinically detectable joint swelling and full-blown PD-positive tenosynovitis; we also assume that tenosynovitis precedes intra-articular synovitis. Given the minimal PD activity of synovial inflammation in patient 10, who carries the same *NOD2* mutation as patient 2 but has never received treatment, we also speculate that active synovial inflammation can spontaneously ameliorate, at least in some patients with Blau syndrome, at some point after their adolescence. These hypotheses, however, need to be confirmed in a large-scale observational study.

The marked improvement in PD scores in patients 5 and 6 after treatment with methotrexate plus infliximab and relatively low total PD scores in patients receiving treatment with methotrexate plus TNF antagonists (patients 3, 4, 7, and 9) support the notion that a methotrexate plus TNF antagonist regimen is efficacious not only for RA [40,41] and JIA [42] but also for arthritis in patients with Blau syndrome [43]. On the other hand, the effectiveness of methotrexate monotherapy was not obvious in our small group of patients. We do not exclude the possible influence of genotypes on the severity of arthritis in patient 3 (D382E) and 4 (R587C), both of which were not included in the previous analysis of the association between genotype and phenotype [6]. A future investigation on the association between *NOD2* genotypes and ultrasound-defined severity of arthritis in a larger number of patients may identify the high-risk genotypes for severe synovial inflammation.

The symmetrical involvement of synovial tissues and the effectiveness of methotrexate plus infliximab in our patients with Blau syndrome support the argument that autoinflammatory diseases such as Blau syndrome and autoimmune diseases such as RA share some phenotypic manifestations and cytokine pathways that maintain arthritis even though the contribution of innate/acquired immunity to pathogenesis is quite different between these two categories of disease [44]. However, our study also confirms the painless nature of arthritis in Blau syndrome, which contrasts with RA. Our patients exhibited surprisingly little tenderness or pain (Table 2) in the presence of active inflammation in both the intra-articular- and teno-synovium (Figures 1 and 2). In addition, the elevation of acute inflammatory responses was infrequent and mild, which is consistent with some of the previous reports [1,14]. These data were reflected in the poor correlations of total ultrasound scores with tender joint count, patients’/parents’ global VAS, ESR, and CRP (Table 3), indicating that these measures do not reflect synovial inflammation in Blau syndrome. The reasons for this absence of canonical features of inflammation in the presence of synovial inflammation, along with the predominance of tenosynovitis, caused by *NOD2* mutations are intriguing research questions in understanding the molecular mechanisms of inflammation.

On the other hand, the swollen joint count tended to correlate with total ultrasound scores (Table 3), suggesting that the swollen joint count is the most reliable conventional measure that can reflect the global severity of synovial inflammation in patients with Blau syndrome. At a joint level, however, active synovial inflammation was frequently detected by ultrasound in joints without swelling (Table 4). These data indicate that clinically detected joint swelling is not as sensitive as ultrasound findings to represent synovial inflammation.

Given the significant improvement of clinical parameters after treatment in patient 5 (Table 2), these measures may reflect the therapeutic effect of potent therapy on arthritis when disease activity is very high. However, in order to prevent structural damage and late-onset functional impairment, treating patients to a minimal disease activity state, which can be only evaluated by ultrasound, may be important. Since the impact of synovial inflammation on structural and functional deterioration in Blau syndrome can be substantially different from that in RA and JIA, longitudinal assessment of arthritis in Blau syndrome using quantitative measures for structural damage (for example, radiographic score) is urgently needed to determine the threshold of acceptable activity of synovial inflammation. In the future, clinical studies to establish optimized treatment strategies (for example, treatment agent, treatment target) for arthritis in Blau syndrome, ultrasound may play a significant role in decreasing the sample size needed by providing better quantification and sensitivity to change.

One of the major limitations of our study is the very small sample size. However, Blau syndrome is a much more homogeneous condition as compared with RA or JIA, especially when the genetic mutation of *NOD2* is confirmed. Also given the quantitative capability and the excellent reproducibility of ultrasonographic assessment of synovial inflammation in the recent reports [22,29,36,37], the comprehensive data obtained in this study are likely to represent the pathophysiology of arthritis in Blau syndrome and provide more reliable information than the mostly descriptive one in the previous reports.

Another major limitation of our study is that ultrasonographic synovial pathologies were graded subjectively due to the lack of currently available standardized measures for children. Although our data support the use of ultrasound in monitoring the disease activity of Blau syndrome, our data cannot be readily generalizable and need to be confirmed in future studies when standardized methods are established. Furthermore, performing the comprehensive ultrasound assessment we employed in this study is not feasible in daily practice. The essential synovial sites to be scanned should be determined not only by the frequency of involvement, but also by the impact on structural and functional deterioration in Blau syndrome. Although we assume that PD signal is more essential than GS synovitis in monitoring disease activity, careful gray-scale assessment is fundamentally important to distinguish between the inflammatory angiogenesis in synovial hypertrophy and the feeding vessels in unossified cartilage in children.

## Conclusions

The detailed anatomical distribution and severity of synovial inflammation revealed by comprehensive ultrasound assessment confirm the frequently involved joints, the predominance of tenosynovitis, and the dissociation between pain and inflammation in the arthritis of Blau syndrome. Our data also give an insight into the treatment response of arthritis in Blau syndrome and demonstrate that musculoskeletal ultrasound can be a potent tool in monitoring the activity of synovial inflammation and in investigating the pathophysiology of arthritis in this rare but archetypical autoinflammatory condition.

## Abbreviations

CHAQ-DI: childhood health assessment questionnaire-disability index; CRP: C-reactive protein; DAS28: disease activity score 28; EOS: early onset sarcoidosis; ESR: erythrocyte sedimentation rate; GS: gray-scale; HAQ-DIL: health assessment questionnaire-disability index; JIA: juvenile idiopathic arthritis; MCP: metacarpophalangeal; MMP-3: metalloproteinase-3; MTP: metatarsophalangeal; NFκB: nuclear factor kappa-light-chain-enhancer of activated B cells; NOD: nucleotide-binding oligomerization domain; NSAID: nonsteroidal anti-inflammatory drug; PD: power Doppler; PIP: proximal interphalangeal; RA: rheumatoid arthritis; RF: rheumatoid factor; TNF: tumor necrosis factor; VAS: visual analogue scale.

## Competing interests

KI, NaK, ST, TK, NoK, RN, HM, and HN have received speaking fees and KI, ST, HM, and HN have also received research grant support from Mitsubishi-Tanabe Pharma Corporation. KI, ST, and HN have received speaking fees and ST and HN have also received research grant support from Takeda Pharmaceutical. KI, ST, HM have received speaking fees and HM has also received research grant support from Pfizer Japan. Other authors declared no competing interests in relation to this work.

## Authors’ contributions

KI, NaK, and ST designed the study. KI performed ultrasound examination. NaK, ST, TN, YI, MT, NO, TS, TY, TK collected the clinical information. NaK, YI, IO, NoK, and RN collected the genetic information. KI, NaK, ST, NS, HM, and HN coordinated the study and analyzed and interpreted the data. KI wrote the first draft of the manuscript. All authors critically revised the first draft and approved the final version.

## Supplementary Material

Additional file 1 **A longitudinal view of the dorsal aspect of the 3**^**rd **^**proximal interphalangeal joint in the right hand.** Severe synovial hypertrophy accompanies severe power Doppler signals. Click here for file

Additional file 2 **A longitudinal view of the flexor tendon of the 3**^**rd **^**finger in the right hand.** Moderate tenosynovial hypertrophy accompanies moderate power Doppler signals. Transverse views of this lesion showed more severe findings. Click here for file

Additional file 3 **An oblique view of the anterior-lateral aspect of the suprapatellar recess in the right knee.** Severe synovial hypertrophy accompanies severe power Doppler signals. Click here for file

Additional file 4 **A longitudinal view of the tibialis anterior tendon in the right ankle.** Severe tenosynovial hypertrophy accompanies moderate to severe power Doppler signals. Click here for file

Additional file 5 **A longitudinal view of the dorsal aspect of the 3**^**rd **^**metatarsophalangeal joint in the right foot.** Mild to moderate synovial hypertrophy accompanies moderate to severe power Doppler signals. Click here for file

Additional file 6 **A longitudinal view of the flexor tendon of the 1**^**st **^**toe in the right foot.** Severe tenosynovial hypertrophy accompanies moderate power Doppler signals. Click here for file
